# Determination of hair cortisol in horses: comparison of immunoassay vs LC-HRMS/MS

**DOI:** 10.1007/s00216-022-04343-6

**Published:** 2022-09-22

**Authors:** Giorgio Saluti, Matteo Ricci, Federica Castellani, Maria Novella Colagrande, Gabriella Di Bari, Michele Podaliri Vulpiani, Francesco Cerasoli, Giovanni Savini, Giampiero Scortichini, Nicola D’Alterio

**Affiliations:** 1 Istituto Zooprofilattico Sperimentale Dell’Abruzzo E del Molise “G. Caporale”, Via Campo Boario, 64100 Teramo, Italy; 2 Department of Veterinary Medical Sciences, University of Bologna, via Tolara di Sopra, 50, 40064 Ozzano dell’Emilia, Bologna Italy

**Keywords:** Cortisol, Hair, Horse, ELISA, LC-HRMS/MS

## Abstract

The measure of hair cortisol concentration (HCC) is becoming an emerging approach to monitor mid-/long-term stress in animals, so it is more and more important to develop accurate and reliable methods. In the light of this, the aim of the present study was to compare mane HCCs of 47 horses with different managements, by means of an immunoassay (ELISA) and liquid chromatography coupled to hybrid high-resolution mass spectrometry (LC-HRMS/MS). After the washing step, the ground hair was extracted with methanol. The extract was evaporated and redissolved in two different aqueous solutions, depending on the detection technique. The methods were validated according to EMA guideline for bioanalytical method validation, in the range 2–50 pg mg^−1^ (ELISA) and 1–100 pg mg^−1^ (LC-HRMS/MS). Satisfactory quantitative performances were obtained for both of the approaches, but this latter demonstrated better precision. The detected concentrations in real samples were encompassing the range 1.3–8.8 pg mg^−1^ and 2.0–17.9 pg mg^−1^ by means of LC-HRMS/MS and ELISA, respectively. Overall, HCCs measured with ELISA technique were 1.6 times higher. The overestimation of immunoassay results might be caused by cross-reactivity phenomena of laboratory reagents and other structurally similar hormones present in the mane.

**Graphical abstract:**

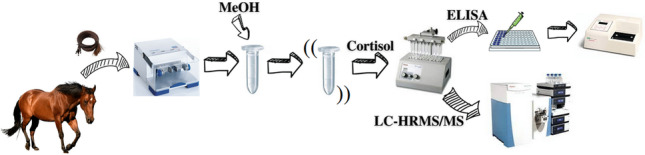

**Supplementary Information:**

The online version contains supplementary material available at 10.1007/s00216-022-04343-6.

## Introduction

The hypothalamic–pituitary–gonadal and hypothalamic–pituitary–adrenal (HPA) axes respond to stress episodes with an increased production of hormones. Particularly, the main glucocorticoid cortisol is secreted by the “ignition” of HPA system and represents a key component of the physiological response to stress. The determination of long-term cortisol levels in matrices such as blood, feces, saliva, and urine is difficult because they are affected by circadian changes, environmental factors, and sudden fluctuations. As a matter of fact, these media permit the adequate assessment of short-term response to stress. On the contrary, hair cortisol concentration represents an accurate measure of mid-/long-term stress. Lipophilic hormones like cortisol are linked into the hair shaft via passive diffusion from the vascular system and secondarily through the tissues surrounding the follicle during the hair growth cycle, although the exact mechanisms of incorporation are not completely known. Moreover, the hair sample is more stable than the traditional matrices at room temperature, and the sampling is pain-free [1]. The potentiality of HCC is having an increasing interest in animal stress and welfare research, indeed several papers investigated the impact of different variables on HCC in wild animals: biological factors (e.g., age, sex, hair color, sampling body region), clinical/medical conditions (e.g., pregnancy, illness, surgeries), environmental factors (e.g., seasonality, light exposure), and finally housing and managements conditions (e.g., relocation, breeding, lifestyle). However, it is worth noting that until now, HCC studies in horses have been scarcely published; in fact, 10 results gave back after inserting of the words “cortisol” and “hair” and “horse” in the PubMed site (Pubmed.gov), but more detailed research on other sources returns a total of 13 papers. The majority of the published studies in animals used immunoassays for HCC measurements, but this technique could not offer high accuracy because of matrix effects and/or hypothetical cross-reactivity phenomena (e.g., related steroid metabolites). Instead, liquid chromatography tandem mass spectrometry (LC–MS/MS), especially high-resolution mass spectrometry (HRMS) permits high selectivity and accuracy [2]. Moreover, quantitative results can be more accurate through the use of isotopically labeled internal standards. The comparison between the two cited techniques was only investigated by Russell and collaborators in human hair [3]. To the best of our knowledge, only our previous study has analyzed hair cortisol in horses with LC-HRMS/MS [4], and no study has so far compared it with immunoassays. The aims of this work were to compare validation method performances and the equine HCCs of real samples, obtained by means of LC-HRMS/MS and an immunoassay (i.e., ELISA) and validation data of the two approaches.

## Materials and methods

### Reagents and materials

The LC–MS grade deionized water, formic acid, and methanol (MeOH) were purchased from Biosolve Chimie (Dieuze, France). The reference material of cortisol and the internal standard (IS) cortisol-d_4_ (9, 11, 12, 12-d_4_) were supplied by Merck KGaA (Darmstadt, Germany).

The stock standard solutions of Cortisol and IS at 1000 and 100 µg mL^−1^, respectively, were dissolved in MeOH and stored at − 20 °C for 6 months. The intermediate ones (1 µg mL^−1^, 100 and 20 ng mL^−1^) were freshly prepared in the dissolving solution or rather a mixture of MeOH/water 50/50 (*v/v*) containing 0.05% formic acid except for the solution at 10 µg mL^−1^ that is stable for 3 weeks in the freezer.

The Cortisol ELISA Kit Item No. 500360 was bought by Cayman Chemical (Michigan, USA).

### Sample preparation

Horse hair samples were prepared as described by Duran et al. [5] with slight modifications. Particularly, the collected sample of about 20 cm length was washed three times with 40 µL methanol/mg hair for about 5 min (per wash) and then dried overnight at room temperature. The first 6 cm of hair next to the root (excluding it) was ground to a fine powder with a Tissue Lyser II (Qiagen, Hilden, Germany) for 5 min at 30 Hz using a stainless steel bead (20 mm diameter). After which, 25 mg of sample was weighed in a 2 mL microcentrifuge and plastic tube and spiked with 12.5 µL of a solution containing the IS a 20 ng mL^−1^. Later, 500 µL of MeOH was added, and the extraction was carried out overnight in a shaker. After centrifugation, two more extractions of 5 min duration were performed. The reunited extracts were filtered with a 0.45 µm PVDF (polyvinylidene fluoride) filter, evaporated, and then redissolved in 100 µL of the dissolving solution (LC-HRMS/MS analysis) or 600 µL of ELISA buffer (ELISA procedure).

### LC-HRMS/MS analysis

The presented method was based on our previous paper [4]. Particularly, chromatography was performed on a Thermo Ultimate 3000 High-Performance Liquid Chromatography system (San Jose, CA, USA) using an Acquity UPLC BEH C18 column (50 × 2.1 mm; 1.7 μm, 130 Å) (Waters, Milford, MA, USA), connected to an Acquity guard column (5 × 2.1 mm). HPLC eluent A was an aqueous solution containing 0.1% (v/v) formic acid, and eluent B was MeOH. The gradient was started with 40% eluent B for 1 min, continued with linear increase to 95% B in 8 min and maintained in this condition for 3 min. The system came back to 40% B in 1 min and was equilibrated for 3 min for a total run time of 15 min. The column compartment and the sample temperature were kept at 30 °C and 16 °C, respectively. The flow rate was 0.25 mL min^−1^, and the injection volume was 10 μL.

A Q‐Orbitrap mass spectrometer (Thermo Scientific, San Jose, CA, USA) was equipped with a heated electrospray ionization (HESI‐II) source. The HESI‐II and capillary temperatures were set at 320 and 300 °C, respectively and the electrospray voltage at 3.20 kV (positive ionization mode). Sheath and auxiliary gas were 35 and 15 arbitrary units, respectively. The mass spectrometer was controlled by Xcalibur 3.0 software (Thermo Fisher Scientific, San Jose, CA, USA). The exact mass of the compounds was calculated using Qualbrowser in Xcalibur 3.0. Instrument calibration was performed for every analytical batch with a direct infusion of LTQ Velos ESI Positive Ion Calibration Solution (Pierce Biotechnology Inc., Rockford, IL, USA). The individual compounds were infused with a syringe through a T union connected to an LC system with a mobile phase flow rate of 0.1 mL min^−1^ (50% eluent A). The product ions were found by increasing the collision energy (CE) using Q‐Exactive Tune 2.3 software (Thermo Fisher Scientific, Waltham, Massachusetts, USA). After choosing the more intense product ions, fragmentation energies were optimized with spiked samples at 1 pg mg^−1^ using the optimized gradient program. All Q Exactive parameters as resolution, automatic gain control (AGC), and injection time (IT) were optimized to improve instrumental signals and selectivity. MS acquisition was performed using full-scan/dd‐MS^2^ experiment: mass range was within m/z 150–1200, resolution set at 70,000 FWHM (*m/z* 200). The AGC was fixed at 3 × 10^6^ ions for a maximum injection time of 300 ms. With regard to MS^2^ acquisition mode, the adduct ion was filtered with an isolation window of m/z 2.0, a resolution, AGC target, and maximum IT were set at 35,000 FWHM (*m/z* 200), 5 × 10^5^, and 140 ms, respectively. The monitored adduct and product ions such as the collision energy are presented in Table 1.

**Table 1:** UHPLC-Q-Orbitrap parameters of cortisol and IS

Analyte	RT (min)	Molecular formula	Adduct	Monoisotopic exact mass (*m/z*)	CE (eV)	Fragment 1 ^1^ (*m/z*)	Fragment 2 ^1^ (*m/z*)
Cortisol	4.5	C_21_H_30_O_5_	[M + Na]^+^	385.1985	30	85.0	288.9
Cortisol-d4	4.5	C_21_H_26_D_4_O_5_	[M + H]^+^	367.2417	-	-	-

^1^Fragments used only for qualitative purposes (ion ratio calculated in product ion scan spectrum)

### ELISA procedure

The commercially available, competitive ELISA is based on the competition between cortisol and cortisol–acetylcholinesterase (AChE) conjugate for the anticortisol antibody binding sites. Particularly, the assay was performed by the addition of cortisol standards/samples, AChE, and the cortisol-specific, mouse monoclonal antibody in the wells. During the incubation (overnight at + 4 °C), the antibody–cortisol (either free or tracer) complex bound to the goat polyclonal anti-mouse IgG captured on the solid phase. After a washing step, the bound enzyme conjugate was revealed thanks to the addition of the Ellman’s reagent (which contains the substrate) producing a yellow product. A microplate reader Benchmark BIO-RAD (Hercules, CA, USA) was used to measure the absorbance at 405 nm. This latter was inversely proportional to the amount of free cortisol present in the well during the incubation. The results were expressed in percentages of the maximum absorbance (*B/B*_*0*_%) using Eq. 1:

1 $$\frac{\mathrm B}{{\mathrm B}_0}\left(\%\right)=\frac{\mathrm{Absorbance}\;\;\mathrm{standard}/\mathrm{sample}}{\mathrm{Absorbance}\;\;\mathrm{at}\;\;\mathrm{zero}\;\;\mathrm{standard}\;\;\mathrm{concentration}}\bullet100$$

The cross-reactivities of steroids in the selected ELISA kit are as follows: cortisol 100%, dexamethasone 15%, prednisolone 4.0%, cortexolone 1.6%, 11-deoxycorticosterone, and 17-hydroxyprogesterone 0.23%, cortisol glucuronide 0.15%, corticosterone 0.14%, cortisone 0.13%, and finally androstenedione, enterolactone, estrone, 17-hydroxypregnenolone, pregnenolone, and testosterone < 0.01% (data from manufacturer).

### Method validation and quality assurance

The validation was carried out following the performance criteria described in EMA guideline on bioanalytical method validation [6]. The calibration curve/linearity has been evaluated in dissolving solution and ELISA buffer. Mane hair used for the validation was collected by a horse outside the 47 real samples. Its measured HCC was 0.5 pg mg^−1^ and 0.9 pg mg^−1^ by means of LC-HRMS/MS and ELISA, respectively. Precision intended as repeatability and within-laboratory reproducibility as well as trueness (recovery), LOD, and LOQ was also assessed in the concentration range encompassing 1–100 pg mg^−1^ (LC-Q-HRMS/MS analysis) and 2–50 pg mg^−1^ (ELISA procedure). In detail, four spiking levels were performed with regard to LC-HRMS/MS approach, 1, 2, 10, and 100 pg mg^−1^, and five spiking levels were carried out for ELISA procedure, 2, 5, 10, 25, and 50 pg mg^−1^. Six replicates for each of them were analyzed out along with a calibration curve prepared in the dissolving solution and ELISA buffer. Each series was repeated on 2 different days for a total of 48 spiked samples, varying time, operator, and calibration status of the LC-HRMS/MS system. Sixty spiked samples were analyzed on 2 days, varying time, and operator, concerning the ELISA procedure. The precision was calculated applying the analysis of variance (ANOVA) at each level. An internal quality control (IQC) was implemented for the analytical batches by adding the cortisol-d4 solution to each sample prior to extraction (only for LC-HRMS/MS analysis). The IS was used with quantitative aims (isotopic dilution). Moreover, a QC sample and at least a spiked QC sample at 10 pg mg^−1^ were analyzed for both two approaches, to verify the absence of a false negative result. It is noteworthy that a procedural blank (PB) was also inserted in each analytical batch during the ELISA procedure, to check the possible presence of interferences. Each real sample was analyzed twice and quantified with a calibration curve in neat solvent, taking into account of the dilution factor.

### Real sample analysis

Hair samples were selected and collected as described by Cerasoli et al. (2022) [4]. Briefly, forty-seven horses belonged to three groups: group 1 (*n* = 16) carried out training activities and flatwork for the Italian State Police of Lazio region 3–4 times a week (the animals stand alone in the boxes with daily access to the paddock), while group 2 (*n* = 16) exercised daily public order activities for the Italian State Police (Lazio region) and lived in boxes with no access to the paddock. Finally, group 3 (*n* = 15) included horses that lived in the wild (Abruzzo region), owned by a farmer. After the collection, the mane hair samples are stable for 2 years at room temperature [7]. They were analyzed by ELISA procedure and LC-HRMS/MS analysis as described before. Data analysis was statistically evaluated after the verification of the normal distribution by means of the Pearson correlation coefficients (regression analysis), as well as Brand–Altman plots, and two paired sample *t* tests (*P* values of < 0.05 were considered significant).

## Results and discussion

### Optimization of LC-HRMS/MS conditions

The chromatographic run was developed testing different gradients with the Acquity UPLC BEH C18 column and MeOH (eluent B)—0.1% (*v/v*) formic acid as mobile phases. A 27-min gradient was initially tested to verify the retention time of cortisol; particularly, it was started with 5% eluent B for 1 min, continued with linear increase to 95% B in 20 min and maintained in this condition for 2 min. The system came back to 5% B in 1 min and was equilibrated for 4 min. After that, the gradient time was gradually reduced, taking into account the good retention of the analyte (i.e., 12.8 min for the initial gradient). The candidate gradients were of 10 and 15 min. The 10-min gradient was initiated with 40% eluent B for 1 min, continued with linear increase to 95% B in 4.5 min. This condition was maintained for 2 min. The system returned to 40% B in 0.5 min and was re-equilibrated for 2.5 min. Instead, the 15 min chromatography started with 40% B for 1 min to linear increase until 95% in 8 min and maintain this condition for 3 min. After returning to initial condition in 1 min, the system was equilibrated for 3 min. Although it is desirable for a short chromatographic run for only two analytes (i.e., cortisol and cortisol-d4), the elution of matrix interferences must be considered for a sample preparation based on a dilute and shoot approach. The 15 min chromatography was finally chosen thanks to the higher instrumental signal of analytes, as a matter of fact; moreover, the full-scan spectrum of spiked samples acquired with the longer gradient appeared “cleaner” (data not shown).

The optimization of MS settings was carried out by direct infusion of a solution of cortisol, followed by the analysis of spiked samples at 1 pg mg^−1^ using the selected gradient program. With regard to this, sodium adduct was chosen thanks to its higher instrumental signal than the protonated molecular ion ([M + H]^+^).

### Method validation and quality control

The validation results obtained with the two techniques are summarized in Table 2 and listed in Table S1 in more detail. The recoveries of cortisol in the spiked samples were determined thanks to a calibration curve (neat solvent) of the analyte for ELISA procedure (sigmoidal curve) and the analyte/IS ratio for LC–MS/MS analysis (isotopic dilution). For this latter, good linearity was observed (deviations of back‐calculated concentration ≤ 15%). It was judged by analyzing curves in the concentration range encompassing the lowest and highest validation level (0.25–25 pg µL^−1^ of cortisol and 2.5 pg µL^−1^ of IS), taking into account the applied dilution factor. The coefficients of variation (CV_r, pooled_ and CV_wR, pooled_) were calculated pooling the single-level CVs after the verification of their substantial equivalence, with regard to the precision. CVs obtained with LC-HRMS/MS technique were lower than the ELISA approach (especially for the spiking levels 2 and 5 pg mg^−1^) (Table S1) because the first technique carries out structural-based measures, contrary to the second one that could be affected by cross-reactivity phenomena. Moreover, cortisol-d4 reduced the ion suppression in LC-HRMS/MS system because any matrix components co-eluting with the analyte will be identical for the IS, allowing the analyte to IS response ratio to compensate this effect. Obviously, this provided a more accurate and rugged method.

**Table 2:** Method precision, recovery, and LOD/LOQ

Parameter	ELISA	LC-HRMS/MS
Mean recovery (%)	110	97
CV_r, pooled_ (%)	18	11
CV_wR, pooled_ (%)	18	13
LOD/LOQ (pg mg^−1^)	2	1

Another issue is that the development/validation of an accurate, quantitative method for endogenous compounds is challenging because of the difficulty to obtain analyte-free authentic matrices as quality control samples. The applied strategy to circumvent this aspect consisted in exceeding the levels of QC samples minimum by a factor of 2 [8] with the spiking experiments**,** so the estimated LOD/LOQ were operative values (Table 2) equal to the lowest validation level. LOD/LOQ of ELISA procedure was higher because of three possible effects such as (i) some other hypothetical, relevant hormones, (ii) chemicals/reagents used during the sample preparation, and (iii) the natural presence of cortisol in the QC samples. Although this last issue can also influence the LC-HRMS/MS approach, procedural blanks (PBs) were carried out applying the ELISA analytical procedure in all respects apart from the addition of the test portion, and interferences were observed. Particularly, during the validation and real sample analysis phases, a PB was inserted within each analytical batch (about 12 samples), and the detected bias value was subtracted to the calculated cortisol concentration. The maximum PB contamination was 3.6 pg mg^−1^.

### Real sample analysis (comparison of cortisol measurements by ELISA and LC-HRMS/MS techniques)

**Fig. 1:**
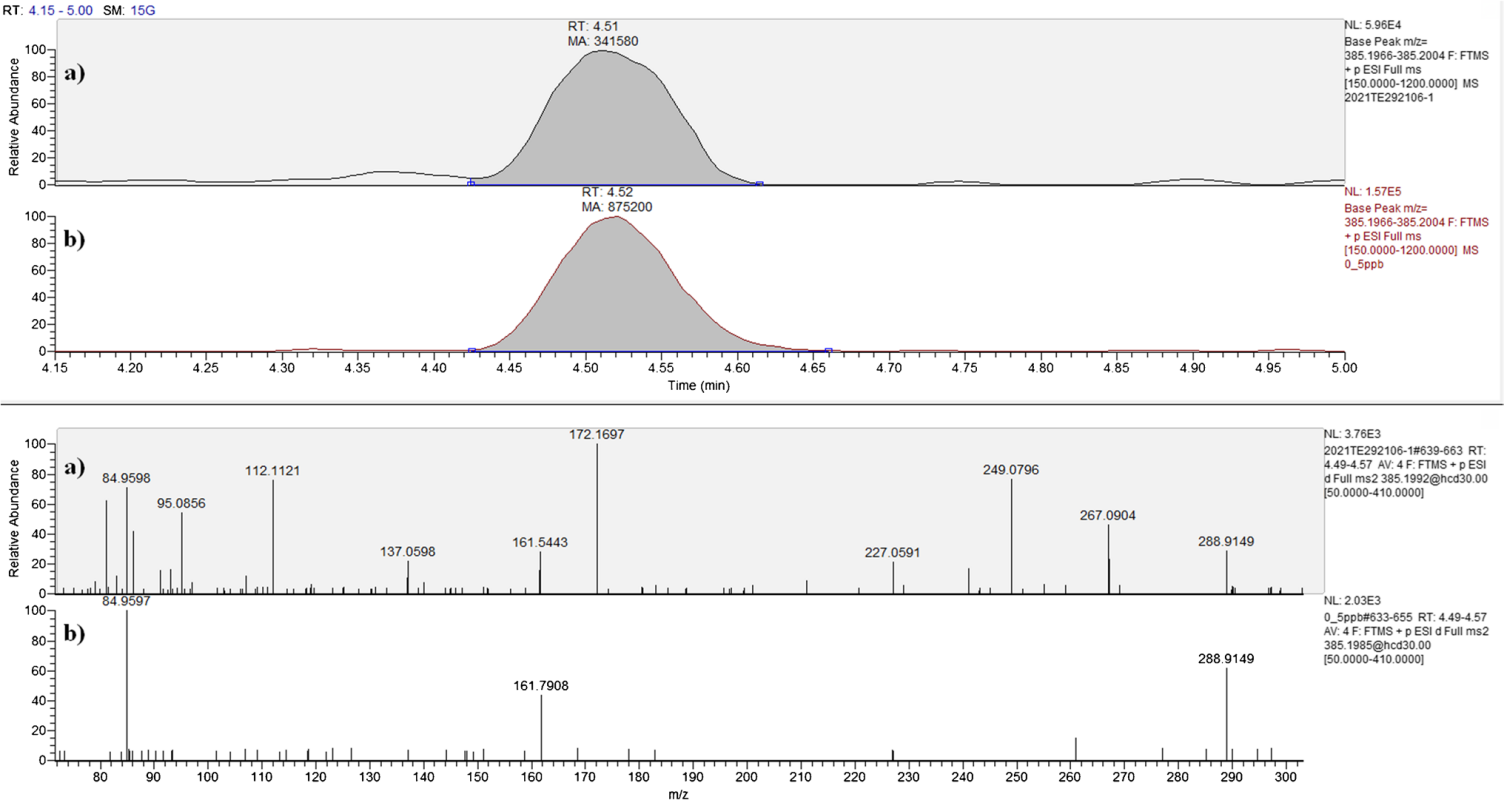
Extract ion chromatograms and product ion spectra of cortisol in mane hair sample at 1.3 pg mg^−1^ (**a**) and in the standard at 0.5 pg µL^−1^ (**b**)

Table 3 lists the detected HCC by means of the two approaches. For the ELISA approach, the cortisol concentration in the samples was calculated by the interpolation of calibration curve obtained with six cortisol solutions prepared in ELISA buffer (0, 0.1, 0.2, 0.625, 2.5, 4 pg µL^−1^), while the HCC detected with LC-HRMS/MS technique was obtained by the interpolation of calibration curve (isotopic dilution) prepared in the dissolving solution at 0.25, 0.5, 2.5 pg µL^−1^ of cortisol and 2.5 pg µL^−1^ of IS. In Fig. 1, the full-scan chromatograms and MS/MS spectra of cortisol in sample 6/group 1 with the lowest detected concentration are shown together with chromatograms and spectra of a standard prepared in dissolving solution. The differences of corrected mean concentrations of ELISA procedure with respect to LC-HRMS/MS analysis were 40%, 35%, and 49%, for hair horse samples of groups 1, 2 and 3, respectively. The correction consisted in the subtraction of the procedural blank contribution. The biases become 46%, 51%, and 46%, not taking into account of the PB contribution. Overall, the mean immunoassay values were 1.6 and 1.9 times higher than LC–MS/MS ones, with or without the subtraction of PB concentrations.

**Table 3:** Detected concentrations of cortisol

Group	ID	ELISA				LC-HRMS/MS	
		Detected concentration (***n*** = 2) (pg mg^−1^)	Corrected*, detected concentration (***n*** = 2) (pg mg^−1^)	Mean detected concentration ± SD (pg mg^−1^)	Corrected*, mean detected concentration ± SD (pg mg^−1^)	Detected concentration (***n*** = 2) (pg mg^−1^)	Mean detected concentration ± SD (pg mg^−1^)
1	1	8.4	7.8	4.6 ± 2.6	4.2 ± 2.6	2.7	2.5 ± 0.7
	2	7.4	6.8			3.4	
	3	8.9	8.3			3.6	
	4	4.1	3.7			1.9	
	5	2.2	1.8			3.1	
	6	2.0	1.2			1.3	
	7	3.3	2.9			2.6	
	8	7.5	7.0			1.9	
	9	4.2	3.6			1.9	
	10	9.3	8.7			3.1	
	11	3.2	2.7			1.5	
	12	2.8	2.3			2.6	
	13	2.9	2.3			2.2	
	14	2.9	2.9			2.3	
	15	2.5	2.5			2.6	
	16	2.8	2.8			2.7	
2	1	5.1	5.1	7.2 ± 3.7	5.4 ± 4.1	1.6	3.5 ± 1.3
	2	2.6	2.6			3.1	
	3	2.4	2.4			1.4	
	4	9.2	9.2			4.2	
	5	9.4	9.4			2.5	
	6	7.6	7.6			2.1	
	7	15.1	15.1			5.6	
	8	9.2	6.2			5.4	
	9	14.8	11.8			3.4	
	10	6.5	2.9			5.6	
	11	7.0	3.4			4.5	
	12	4.6	1.0			3.5	
	13	6.6	3.0			3.2	
	14	5.7	2.1			3.5	
	15	4.8	1.2			2.9	
	16	5.3	3.6			4.2	
3	1	7.9	6.3	9.7 ± 3.5	8.5 ± 5.7	8.8	5.2 ± 1.4
	2	5.7	4.1			3.8	
	3	6.4	4.8			4.5	
	4	5.8	4.1			3.7	
	5	6.4	4.8			5.3	
	6	6.4	5.5			5.3	
	7	17.9	17.0			6.6	
	8	14.2	13.3			6.9	
	9	10.1	9.2			3.8	
	10	9.3	8.4			4.3	
	11	12.5	11.6			4.3	
	12	11.5	10.6			5.4	
	13	10.0	9.1			6.3	
	14	11.9	11.0			4.9	
	15	9.0	8.1			4.4	

*Concentration of the procedural blank analyzed in each analytical batch

**Fig. 2:**
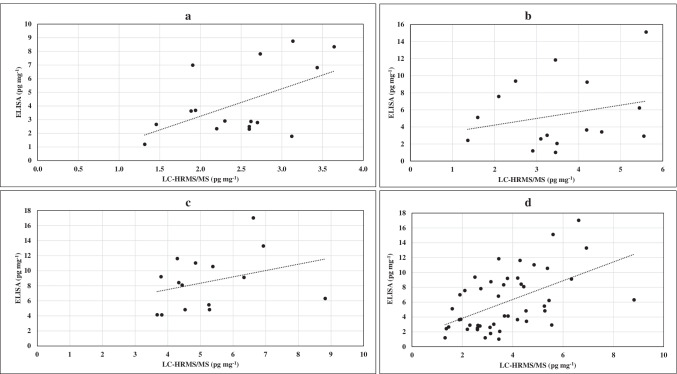
Scatterplot and linear regression lines of concentrations obtained via ELISA and LC-HRMS/MS for groups 1 (**a**), 2 (**b**), and 3 (**c**) and for all the data (**d**)

**Fig. 3:**
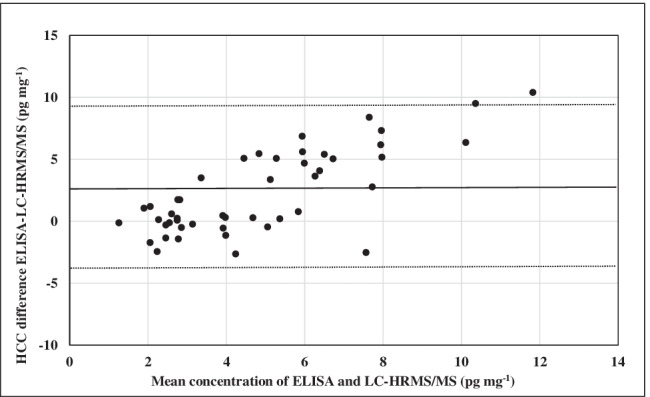
Bland–Altman plot with a mean bias (black, solid line) of 2.3 pg mg^−1^ and 95% limits of agreement (black, dashed lines) of − 4.4 and 9.0 pg mg^−1^

The correlations between cortisol concentrations have been evaluated using a scatterplot, considering the linear regression lines (Fig. 2). The total data obtained by the two approaches, taking into account the corrected concentrations of ELISA procedure, did not show a good Pearson correlation coefficient (*r* = 0.5232), and two paired samples *t* test (*t* value = 4.662, *p* = 2.7 × 10^−5^) indicated significant difference between the two populations (*p* < 0.05). In detail, *r* values were equal to 0.5254, 0.2494, and 0.3200 for groups 1, 2, and 3, respectively, while *p* values were 0.008, 0.081, and 0.0029. Interestingly, data of the group 2 belonged to the same population. Bland–Altman plot of total data (Fig. 3) also demonstrated limited agreement between the two approaches with an increasing difference for higher detected concentrations.

In the light of these results, higher HCCs were detected using the ELISA procedure probably due to the aforementioned cross-reactivity phenomena that could provide an overestimation of the found value.

### Overview of the methods for HCC determination

In Table 4, an overview of the main published methods for the determination of horse hair cortisol is reported. The first paper, published in 2012 by Comin et al. [9], proposed a radioimmunoassay (RIA) method to measure HCC in withers foals at birth and until 2 months. Particularly, the cortisol level decreased with time and varied markedly between the animals. Two years later, Montillo et al. (2014) [10] applied a RIA method to a large group of foals (i.e., 219) to investigate the effects of environmental conditions, age (from the birth to 30 days of life), and sex. Later, Duran and coworkers [5] developed and used an ELISA method to analyze hair of 38 mares and yearlings for studying the variables pregnancy, surgical castration, the sampling body regions, and the cortisol concentration along the hair. In 2019, Placci et al. [11] evaluated the HCC, hair dehydroepiandrosterone (DHEA) (RIA methods), and some blood parameters in 47 sporting horses managed with two different typologies (i.e., conventional and natural ones). This latter seems to comply more with the physiological need of horses. In the same year, an illness condition such as the equine squamous gastric disease (ESGD) was evaluated in terms of stress by Prinsloo and coworkers [12] using an ELISA method. To the best of our knowledge, in the following years, only other seven manuscripts have been published [1, 13–18]. With regard to sampling body region, mane and withers have been chosen more often [10, 12, 15, 19]. Generally, the washing procedure for removing external contamination is based on isopropanol, but three papers did not furnish this information [10, 15, 16]. A common aspect of all the reviewed sample preparation protocols is the extracting solvent which is MeOH. With regard to the homogenization procedure, the most common strategy is the grinding as it assures greater contact surface with the extracting solvent than the cutting and mincing. Nevertheless, five of thirteen papers propose the cutting [9, 11, 12, 16], while Montillo et al. (2014) [10] did not provide the procedure. The limits of detection (LOD) of the published methods were at ppb or sub-ppb levels, but often they were not provided. Particularly, only six papers provided the LODs referred to the sample [9, 11, 14, 16, 17]. The detected HCC levels were generally very different, probably also because of the known limitations of immunoassays, considering no study analyzed PBs to evaluate the interferences. The range of provided HCC was from 1.3 pg mg^−1^ (this work) to 510 pg mg^−1^ [17]. In a few, other cases, the HCCs were only reported in the graphs or figures [13, 16]. It is noteworthy that the detection/quantitation of horsehair cortisol has been carried out almost exclusively with immunoassay techniques (i.e., EIA and RIA) except for the previous published paper [4].

**Table 4:** Overview of the methods for the determination of hair cortisol in horses

Involved animals (number^§^)	Investigated variables	Hair sampling locations	Homogenization	Washing	Extraction	Technique	LOD (pg mg^−1^)	Detected concentration (min–max or mean–SD–mean + SD) (pg mg^−1^)	Reference
Horse foals (102)	Age	Withers	Cutting (1 cm)	Isopropanol (once)	MeOH	RIA	0.21	4.57–156.51	Comin et al. (2012) [9]
Horse foals (219)	Age, light conditions, rainfall, sex, temperature	Withers	NP	NP	MeOH	RIA	NP*	35.17–60.37 (mean values)	Montillo et al. (2014) [10]
Mares and yearlings (38)	Body region, distribution along the hair, pregnancy, surgical castration	Mane (midneck region), tail	Grinding	MeOH (three times)	MeOH	ELISA	NP	2.4–3.3	Duran et al. (2017) [5]
Sporting horses (47)	Conventional and natural management	Mane	Cutting (1–3 mm)	Isopropanol (once)	MeOH	RIA	0.26	0.19–0.90	Placci et al. (2019) [11]
Horses “teaching heard” (25)	ESGD, sex (mares, geldings),	Withers	Cutting (< 2 mm)	Isopropanol (once)	MeOH	ELISA	NP	2.5–10.6	Prinsloo et al. (2019) [12]
Horses (9)	Body region, PPID, seasonality	Jugular groove, mandible, neck, sternum	Grinding	Water (twice), isopropanol (once)	MeOH	ELISA	NP	NP**	Banse et al. (2020) [13]
Pure Spanish stallions (5)	Hair growth, relocation, seasonality	Ventral abdomen	Grinding	Isopropanol (three times)	MeOH	ELISA	0.32	1.83–9.33	Gardela et al. (2020) [14]
Horses (153)	Body region, management, personality	Mane, withers	Grinding	NP	MeOH	ELISA	NP	4.2–14.0 (median values)	Sauveroche et al. (2020) [15]
Horses (40)	Management, state of health (healthy, BPHs)	NP	Cutting	NP	MeOH	RIA	0.26	NP	Arena et al. (2021) [16]
Leisure horses (19)	Age, hair color, management, seasonality, sex	Neck	Grinding	Water (twice), isopropanol (once)	MeOH	ELISA	0.05672	130–510	Mazzola et al. (2021) [17]
Pure-breed Menorca stallions (10)	Seasonality	Sternum	Grinding	Isopropanol (three times)	MeOH	EIA	NP*	3.4–6.3	Olvera – Maneu et al. (2021) [1]
Horses (31)	Body region, hair color, sex	Chest, saddles, thoracic Fetlocks, mane behind the ears, tail strand, withers	Cutting (1–3 mm)	Isopropanol (three times)	MeOH	ELISA	NP*	5.52–29.68	Lelláková et al. (2022) [18]
Horses (47)	Management	Mane	Grinding	MeOH (three times)	MeOH	LC-HRMS/MS	1.0 (LC-HRMS/MS)	1.3–8.8 (LC-HRMS/MS)	Cerasoli et al. (2022) [4]
Horses (47)	Management	Mane	Grinding	MeOH (three times)	MeOH	LC-HRMS/MS ELISA	1.0 (LC-HRMS/MS) 2.0 (ELISA)	1.3–8.8 (LC-HRMS/MS) 2.0–17 (ELISA)	This work

*NP*, not provided; *PPID*, pituitary pars intermedia dysfunction; *ESGD*, equine squamous gastric disease; *EIA*, enzyme immunoassay; *ELISA*, enzyme-linked immunosorbent assay; *RIA*, radioimmunoassay; *LC-HRMS/MS*, liquid chromatography tandem high-resolution mass spectrometry; *BPHs*, horses displaying behavioral pathologies

^§^Maximum number of animals enrolled in the experiments

*LOD expressed as sensitivity in solvent

**Some or all the detected concentrations were only reported in the graphs

Table S2 gathers a survey of the main researches for the determination of hair cortisol in non-domestic animals, since 2005, for a total of 54 reviewed papers [19–72]. To the best of our knowledge, currently, the bovine is the most studied species in terms of HCC with 19 published papers followed by swine (11), ovine (9), bears (7), non-human primates (5), and other less investigated species like caribou and reindeer, chipmunks, hares, lynx, marmots, and minks.

In 2006, Davenport and coworkers [20] published the first paper about this concern: briefly, they evaluated the effects of relocation and several washing procedures measuring HCC of 20 rhesus macaques.

Among the reviewed papers, several factors have been evaluated in correlation to HCC: the most investigated variable is the sampling body region of tested animals [22, 23, 25, 30–33, 36, 40, 42, 46, 51, 52, 57, 60, 72], and the detected concentrations may be highly variable; therefore, it seems reasonable that sampling be standardized to a single body region of each species. Again, the seasonality has been considered by some papers [19, 24, 31, 39, 46, 57, 60, 66]. By way of example, in 2014, Bacci and coworkers [24] applied a RIA method to 30 sows, and the season of the year was observed to have an effect with the lowest HCC measured during the summer. Later, Heimburge et al. (2020) [57] correlated the seasonality and HCC on 75 sows and 72 cows. Only for cattle, there was an influence with higher HCC in winter samples compared with summer samples. One year later, the same variable was studied [60] on 13 polar bears from 12 zoos, and no seasonal hair cortisol variation was observed. Management typologies were selected at times, as in the case of Comin et al. (2011) [19] that examined HCC produced in response to change from indoor winter to summer grazing conditions (high mountain) in 83 dairy cows applying a RIA method. HCC was also related to somatic cell count (SSC), body condition score (BCS), and milk yield and results evidenced a beneficial impact of grazing on the health of animals although associated to a worsening BCS, increased SCC, a reduction of milk yield during the grazing, and a slight rise of HCC during the transition followed by its stabilization. The HCC comparison between different management has been described in our previous paper [4]; briefly, lower level of cortisol was detected in horses that carried out public order activity and had access to the paddock, followed by animals belonging to other two groups. The free-ranging horses provided higher HCC, probably because they lived in more stressful conditions (e.g., fear of being predated, long distance covered in the wild).

Six years later, Peric et al. (2017) [41] evaluated the allostatic load of 27 and 18 dairy cows managed indoor in tie-stall barn and grazing on high mountain pasture, respectively. The authors monitored HCCs, hair DHEA concentrations, and their ratio using two RIA methods, and interestingly, fluctuations and higher stress level were recorded for cows allowed to graze. According to the authors, these results could depend on the movement of animals to a pasture with lower nutritional value and on an increment of physical activity.

In most cases, the homogenization of hair samples was carried out through the grinding, the washing protocol based on isopropanol, and the cortisol extraction on methanol. The detection was carried out in almost all reviewed papers with EIA and RIA methods, except for this work, Binz et al., Braun et al. [35, 39, 49], Hein and coworkers [60], and Cerasoli et al. [4] for which liquid chromatography tandem mass spectrometry and LC-HRMS/MS techniques were applied.

## Conclusions

The developed and validated methods for the determination of cortisol in equine hair samples by means of ELISA and LC-HRMS/MS were fitted for purpose, according to the EMA guideline on bioanalytical method validation [6].

The LC-HRMS/MS method appeared more accurate taking into account of the validation performances, so its application to real sample analysis seems to be more reliable than the ELISA procedure. Although LC–MS/MS technique is currently considered as the gold standard for the quali–quantitative analysis in many research fields, it requires initial higher cost for the instrumentation and more technical expertise than the immunoassays.

Taking into account the limitations of the immunoassay methods and the absence of commercial proficiency tests, the detected concentrations could be affected by a bias, so on the one hand, the comparative analysis improves the knowledge/interpretation of animal welfare, but on the other, absolute values of concentrations must be carefully evaluated.

This work could also offer the perspective to measure HCC on animals by means of LC-HRMS/MS (or more broadly LC–MS/MS) technique to establish threshold values of stress-level exposure, strongly improving the knowledge of animal welfare and giving advice to farmers in order to solve welfare problems.

The use of high-resolution mass spectrometry with accurate mass full-spectrum data could even provide the possibility to elucidate the molecular formula and the chemical structure of currently unknown metabolites or potential biomarkers, looking for a not too distant future.

## Supplementary Information

Below is the link to the electronic supplementary material.

Supplementary file1 (DOCX 32.8 KB)

## References

[1] Olvera-maneu S, Carbajal A, Gardela J. Hypothalamic–pituitary–adrenal and hypothalamic–pituitary–gonadal axes activity: exploring the influence of seasonality. 2021; 1–10 10.3390/ani11082202 PMC8388521 34438659

[2] Sauer FJ, Gerber V, Frei S, Bruckmaier RM, Groessl M (2020). Salivary cortisol measurement in horses: immunoassay or LC-MS/MS?. Domest Anim Endocrinol.

[3] Russell E, Kirschbaum C, Laudenslager ML, Stalder T, De Rijke Y, Van Rossum EFC, Van Uum S, Koren G (2015). Toward standardization of hair cortisol measurement: results of the first international interlaboratory round robin. Ther Drug Monit.

[4] Cerasoli F, Vulpiani MP, Saluti G, Conte A, Ricci M, Savini G, Alterio ND (2022). Assessment of welfare in groups of horses with different management, environments and activities by measuring cortisol in horsehair, using liquid chromatography coupled to hybrid orbitrap high-resolution mass spectrometry. Animals.

[5] Duran MC, Janz DM, Waldner CL, Campbell JR, Marques FJ (2017). Hair cortisol concentration as a stress biomarker in horses: associations with body location and surgical castration. J Equine Vet Sci.

[6] EMA (1922) Guideline on bioanalytical method validation. 44:1–23

[7] Yamanashi Y, Teramoto M, Morimura N, Hirata S, Suzuki J, Hayashi M, Kinoshita K, Murayama M, Idani G (2016). Analysis of hair cortisol levels in captive chimpanzees: effect of various methods on cortisol stability and variability. MethodsX.

[8] Päpke O, Fürst P, Herrmann T (2004). Determination of polybrominated diphenylethers (PBDEs) in biological tissues with special emphasis on QC/QA measures. Talanta.

[9] Comin A, Veronesi MC, Montillo M, Faustini M, Valentini S, Cairoli F, Prandi A (2012). Hair cortisol level as a retrospective marker of hypothalamic-pituitary-adrenal axis activity in horse foals. Vet J.

[10] Montillo M, Comin A, Corazzin M, Peric T, Faustini M, Veronesi MC, Valentini S, Bustaffa M, Prandi A (2014). The effect of temperature, rainfall, and light conditions on hair cortisol concentrations in newborn foals. J Equine Vet Sci.

[11] Placci M, Marliani G, Sabioni S, Gabai G, Mondo E, Borghetti P, De Angelis E, Accorsi PA (2020). Natural horse boarding vs traditional stable: a comparison of hormonal, hematological and immunological parameters. J Appl Anim Welf Sci.

[12] Prinsloo M, Hynd P, Franklin S, Weaver S, van den Boom R (2019). Hair cortisol concentration is inversely related to the severity of equine squamous gastric disease. Vet J.

[13] Banse HE, Getachew F, Levy M, Smits J (2020). Influence of season and pituitary pars intermedia dysfunction on hair cortisol concentration in horses. Domest Anim Endocrinol.

[14] Gardela J, Carbajal A, Tallo-Parra O, Olvera-Maneu S, Álvarez-Rodríguez M, Jose-Cunilleras E, López-Béjar M (2020) Temporary relocation during rest periods: relocation stress and other factors influence hair cortisol concentrations in horses. Animals 10:10.3390/ani10040642 10.3390/ani10040642 PMC7222751 32276388

[15] Sauveroche M, Henriksson J, Theodorsson E, Svensson Holm AC, Roth LSV (2020). Hair cortisol in horses (Equus caballus) in relation to management regimes, personality, and breed. J Vet Behav.

[16] Arena I, Marliani G, Sabioni S, Gabai G, Bucci D, Accorsi PA (2021). Assessment of horses’ welfare: behavioral, hormonal, and husbandry aspects. J Vet Behav.

[17] Mazzola SM, Colombani C, Pizzamiglio G, Cannas S, Palestrini C, Costa ED, Gazzonis AL, Bionda A, Crepaldi P. Do you think I am living well? A four-season hair cortisol analysis on leisure horses in different housing and management conditions. Animals. 2021;11:10.3390/ani11072141 10.3390/ani11072141 PMC8300697 34359269

[18] Lelláková M, Lešková L, Florián M, Mesarčová L, Skurková L, Peťková B, Takáčová D, Kottferová J (2022) Cortisol concentration in horsehair and its relationship to body location, coat colour, and gender. J Equine Vet Sci 115:10.1016/j.jevs.2022.104010 10.1016/j.jevs.2022.104010 35577110

[19] Comin A, Prandi A, Peric T, Corazzin M, Dovier S, Bovolenta S (2011). Hair cortisol levels in dairy cows from winter housing to summer highland grazing. Livest Sci.

[20] Davenport MD, Tiefenbacher S, Lutz CK, Novak MA, Meyer JS (2006). Analysis of endogenous cortisol concentrations in the hair of rhesus macaques. Gen Comp Endocrinol.

[21] Peric T, Comin A, Corazzin M, Montillo M, Cappa A, Campanile G, Prandi A (2013). Short communication: hair cortisol concentrations in Holstein-Friesian and crossbreed F1 heifers. J Dairy Sci.

[22] Terwissen CV, Mastromonaco GF, Murray DL (2013). Influence of adrenocorticotrophin hormone challenge and external factors (age, sex, and body region) on hair cortisol concentration in Canada lynx (Lynx canadensis). Gen Comp Endocrinol.

[23] Yamanashi Y, Morimura N, Mori Y, Hayashi M, Suzuki J (2013). Cortisol analysis of hair of captive chimpanzees (Pan troglodytes). Gen Comp Endocrinol.

[24] Bacci ML, Nannoni E, Govoni N, Scorrano F, Zannoni A, Forni M, Martelli G, Sardi L (2014). Hair cortisol determination in sows in two consecutive reproductive cycles. Reprod Biol.

[25] Burnett TA, Madureira AML, Silper BF, Nadalin A, Tahmasbi A, Veira DM, Cerri RLA (2014). Short communication: factors affecting hair cortisol concentrations in lactating dairy cows. J Dairy Sci.

[26] Cattet M, Macbeth BJ, Janz DM, Zedrosser A, Swenson JE, Dumond M, Stenhouse GB (2014). Quantifying long-term stress in brown bears with the hair cortisol concentration: a biomarker that may be confounded by rapid changes in response to capture and handling. Conserv Physiol.

[27] Dettmer AM, Novak MA, Meyer JS, Suomi SJ (2014). Population density-dependent hair cortisol concentrations in rhesus monkeys (Macaca mulatta). Psychoneuroendocrinology.

[28] Ghassemi Nejad J, Lohakare JD, Son JK, Kwon EG, West JW, Sung KI (2014). Wool cortisol is a better indicator of stress than blood cortisol in ewes exposed to heat stress and water restriction. Animal.

[29] Martelli G, Sardi L, Stancampiano L, Govoni N, Zannoni A, Nannoni E, Forni M, Bacci ML (2014). A study on some welfare-related parameters of hDAF transgenic pigs when compared with their conventional close relatives. Animal.

[30] Mastromonaco GF, Gunn K, McCurdy-Adams H, Edwards DB, Schulte-Hostedde AI (2014). Validation and use of hair cortisol as a measure of chronic stress in eastern chipmunks (Tamias striatus). Conserv Physiol.

[31] Macbeth BJ, Cattet MRL, Stenhouse GB, Gibeau ML, Janz DM (2010). Hair cortisol concentration as a noninvasive measure of long-term stress in free-ranging grizzly bears (Ursus arctos): considerations with implications for other wildlife. Can J Zool.

[32] Carlitz EHD, Kirschbaum C, Miller R, Rukundo J, van Schaik CP (2015). Effects of body region and time on hair cortisol concentrations in chimpanzees (Pan troglodytes). Gen Comp Endocrinol.

[33] Stubsjøen SM, Bohlin J, Dahl E, Knappe-Poindecker M, Fjeldaas T, Lepschy M, Palme R, Langbein J, Ropstad E (2015). Assessment of chronic stress in sheep (part I): the use of cortisol and cortisone in hair as non-invasive biological markers. Small Rumin Res.

[34] Tallo-Parra O, Manteca X, Sabes-Alsina M, Carbajal A, Lopez-Bejar M (2014). Hair cortisol detection in dairy cattle by using EIA: protocol validation and correlation with faecal cortisol metabolites. Animal.

[35] Binz TM, Braun U, Baumgartner MR, Kraemer T (2016). Development of an LC–MS/MS method for the determination of endogenous cortisol in hair using 13C3-labeled cortisol as surrogate analyte. J Chromatogr B Anal Technol Biomed Life Sci.

[36] Fourie NH, Brown JL, Jolly CJ, Phillips-Conroy JE, Rogers J, Bernstein RM (2016). Sources of variation in hair cortisol in wild and captive non-human primates. Zoology.

[37] Peric T, Comin A, Corazzin M, Montillo M, Canavese F, Stebel M, Prandi A (2017). Relocation and hair cortisol concentrations in New Zealand white rabbits. J Appl Anim Welf Sci.

[38] Salaberger T, Millard M, El MS, Möstl E, Grünberger V, Krametter-Frötscher R, Wittek T, Palme R (2016). Influence of external factors on hair cortisol concentrations. Gen Comp Endocrinol.

[39] Braun U, Michel N, Baumgartner MR, Hässig M, Binz TM (2017). Cortisol concentration of regrown hair and hair from a previously unshorn area in dairy cows. Res Vet Sci.

[40] Casal N, Manteca X, Peña LR, Bassols A, Fàbrega E (2017). Analysis of cortisol in hair samples as an indicator of stress in pigs. J Vet Behav Clin Appl Res.

[41] Peric T, Corazzin M, Romanzin A, Bovolenta S, Prandi A, Montillo M, Comin A (2017). Cortisol and DHEA concentrations in the hair of dairy cows managed indoor or on pasture. Livest Sci.

[42] Ashley NT, Barboza PS, Macbeth BJ, Janz DM, Cattet MRL, Booth RK, Wasser SK (2011). Glucocorticosteroid concentrations in feces and hair of captive caribou and reindeer following adrenocorticotropic hormone challenge. Gen Comp Endocrinol.

[43] Esposito L, Auletta L, Ciani F, Pelagalli A, Pasolini MP, Lamagna B, Piscopo N, Amici A. Hair cortisol levels in captive brown hare (Lepus europaeus): potential effect of sex, age, and breeding technology. Eur J Wildl Res. 2017; 63:10.1007/s10344-017-1121-6

[44] Ghassemi Nejad J, Kim BW, Lee BH, Il SK (2017). Coat and hair color: hair cortisol and serotonin levels in lactating Holstein cows under heat stress conditions. Anim Sci J.

[45] Kroshko T, Kapronczai L, Cattet MRL, Macbeth BJ, Stenhouse GB, Obbard ME, Janz DM (2017). Comparison of methanol and isopropanol as wash solvents for determination of hair cortisol concentration in grizzly bears and polar bears. MethodsX.

[46] Acker M, Mastromonaco G, Schulte-Hostedde AI (2018). The effects of body region, season and external arsenic application on hair cortisol concentration. Conserv Physiol.

[47] Carroll GA, Boyle LA, Hanlon A, Palmer MA, Collins L, Griffin K, Armstrong D, O’Connell NE (2018). Identifying physiological measures of lifetime welfare status in pigs: exploring the usefulness of haptoglobin, C-reactive protein and hair cortisol sampled at the time of slaughter. Ir Vet J.

[48] Bergamin C, Comin A, Corazzin M, Faustini M, Peric T, Scollo A, Gottardo F, Montillo M, Prandi A (2019). Cortisol, DHEA, and sexual steroid concentrations in fattening pigs’ hair. Animals.

[49] Braun U, Wiest A, Lutz T, Riond B, Stirn M, Hilbe M, Baumgartner MR, Binz TM (2019). Hair cortisol concentration in veal calves reared under two different welfare production labels. Res Vet Sci.

[50] Dulude-de Broin F, Côté SD, Whiteside DP, Mastromonaco GF (2019). Faecal metabolites and hair cortisol as biological markers of HPA-axis activity in the Rocky mountain goat. Gen Comp Endocrinol.

[51] Fürtbauer I, Solman C, Fry A (2019). Sheep wool cortisol as a retrospective measure of long-term HPA axis activity and its links to body mass. Domest Anim Endocrinol.

[52] Ghassemi Nejad J, Lee BH, Kim JY, Kim BW, Chemere B, Park KH, Il SK (2019). Comparing hair cortisol concentrations from various body sites and serum cortisol in Holstein lactating cows and heifers during thermal comfort zone. J Vet Behav.

[53] Bechshøft T, Sonne C, Dietz R, Born EW, Novak MA, Henchey E, Meyer JS (2011). Cortisol levels in hair of East Greenland polar bears. Sci Total Environ.

[54] Ghassemi Nejad J, Park KH, Forghani F, Lee HG, Lee JS, Il SK (2020). Measuring hair and blood cortisol in sheep and dairy cattle using RIA and ELISA assay: a comparison. Biol Rhythm Res.

[55] Sawyer G, Webster D, Narayan E (2019). Measuring wool cortisol and progesterone levels in breeding maiden Australian merino sheep (Ovis aries). PLoS ONE.

[56] Sharma A, Umapathy G, Kumar V, Phillips CJC (2019). Hair cortisol in sheltered cows and its association. Animals.

[57] Heimbürge S, Kanitz E, Tuchscherer A, Otten W (2020). Within a hair’s breadth – factors influencing hair cortisol levels in pigs and cattle. Gen Comp Endocrinol.

[58] López-Arjona M, Tecles F, Mateo S V., Contreras-Aguilar MD, Martínez-Miró S, Cerón JJ, Martínez-Subiela S. Measurement of cortisol, cortisone and 11β-hydroxysteroid dehydrogenase type 2 activity in hair of sows during different phases of the reproductive cycle. Vet J. 2020; 259–260:10.1016/j.tvjl.2020.105458 10.1016/j.tvjl.2020.105458 32553232

[59] Nejad JG, Lee BH, Kim JY, Chemere B, Sung KI, Lee HG (2021). Effect of alpine grazing on plasma and hair cortisol, serotonin, and DHEA in dairy cows and its welfare impact. Domest Anim Endocrinol.

[60] Hein A, Baumgartner K, von Fersen L, Bechshoft T, Woelfing B, Kirschbaum C, Mastromonaco G, Greenwood AD, Siebert U (2021). Analysis of hair steroid hormones in polar bears (Ursus maritimus) via liquid chromatography–tandem mass spectrometry: comparison with two immunoassays and application for longitudinal monitoring in zoos. Gen Comp Endocrinol.

[61] Lagoda ME, O’Driscoll K, Marchewka J, Foister S, Turner SP, Boyle LA (2021). Associations between skin lesion counts, hair cortisol concentrations and reproductive performance in group housed sows. Livest Sci.

[62] Morgan L, Meyer J, Novak S, Younis A, Ahmad WA, Raz T (2021). Shortening sow restraint period during lactation improves production and decreases hair cortisol concentrations in sows and their piglets. Animal.

[63] Otten W, Bartels T, Heimbürge S, Tuchscherer A, Kanitz E (2021). The dark side of white hair? Artificial light irradiation reduces cortisol concentrations in white but not black hairs of cattle and pigs. Animal.

[64] González-de-la-Vara M del R, Valdez RA, Lemus-Ramirez V, Vázquez-Chagoyán JC, Villa-Godoy A, Romano MC. Effects of adrenocorticotropic hormone challenge and age on hair cortisol concentrations in dairy cattle. Can J Vet Res. 2011; 75:216-221 PMC3122973 22210998

[65] Probo M, Peric T, Fusi J, Prandi A, Faustini M, Veronesi MC (2021). Hair cortisol and dehydroepiandrosterone sulfate concentrations in healthy beef calves from birth to 6 months of age. Theriogenology.

[66] Shi R, Dou J, Liu J, Sammad A, Luo H, Wang Y, Guo G, Wang Y (2021). Genetic parameters of hair cortisol as an indicator of chronic stress under different environments in Holstein cows. J Dairy Sci.

[67] Weaver SJ, Hynd PI, Ralph CR, Hocking Edwards JE, Burnard CL, Narayan E, Tilbrook AJ (2021). Chronic elevation of plasma cortisol causes differential expression of predominating glucocorticoid in plasma, saliva, fecal, and wool matrices in sheep. Domest Anim Endocrinol.

[68] Wiechers DH, Brunner S, Herbrandt S, Kemper N, Fels M (2021). Analysis of hair cortisol as an indicator of chronic stress in pigs in two different farrowing systems. Front Vet Sci.

[69] Macbeth BJ, Cattet MRL, Obbard ME, Middel K, Janz DM (2012). Evaluation of hair cortisol concentration as a biomarker of long-term stress in free-ranging polar bears. Wildl Soc Bull.

[70] Comin A, Peric T, Corazzin M, Veronesi MC, Meloni T, Zufferli V, Cornacchia G, Prandi A (2013). Hair cortisol as a marker of hypothalamic-pituitary-adrenal axis activation in Friesian dairy cows clinically or physiologically compromised. Livest Sci.

[71] Malcolm KD, McShea WJ, Van Deelen TR, Bacon HJ, Liu F, Putman S, Zhu X, Brown JL (2013). Analyses of fecal and hair glucocorticoids to evaluate short- and long-term stress and recovery of Asiatic black bears (Ursus thibetanus) removed from bile farms in China. Gen Comp Endocrinol.

[72] Moya D, Schwartzkopf-Genswein KS, Veira DM (2013). Standardization of a non-invasive methodology to measure cortisol in hair of beef cattle. Livest Sci.

