# Supplementary material for: Determination of hair cortisol in horses: comparison of immunoassay vs LC-HRMS/MS
Source: Anal Bioanal Chem. 2022 Sep 22;414(28):8093–105. doi: 10.1007/s00216-022-04343-6 (PMC9613578; doi:10.1007/s00216-022-04343-6)
Supplement: Supplementary file 1 — Supplementary file1 (DOCX 32.8 KB) [file 216_2022_4343_MOESM1_ESM.docx]

**Determination of hair cortisol in horses. Comparison of immunoassay vs LC-HRMS/MS**

Giorgio Saluti^1^*, Matteo Ricci^1§^, Federica Castellani^1§^, Maria Novella Colagrande^1^, Gabriella Di Bari^2^, Michele Podaliri Vulpiani^1^, Francesco Cerasoli^1^, Giovanni Savini^1^, Giampiero Scortichini^1^, Nicola D’Alterio^1^

^1^Istituto Zooprofilattico Sperimentale dell’Abruzzo e del Molise “G. Caporale”, via Campo Boario, 64100, Teramo (Italy)

^2^University of Bologna, Department of Veterinary Medical Sciences, via Tolara di Sopra, 50, 40064, Ozzano dell’Emilia, Bologna (Italy)

*corresponding author

^§^these authors contributed equally

**Supplementary Information**

**Table S1** Validation data obtained at each level (n = 6) within 2 days.

| **ELISA procedure** | | | |
| --- | --- | --- | --- |
| **Spiking level (pg mg^-1^)** | **Recovery (%)** | **CV_r_ (%)** | **CV_wR_ (%)** |
| 2 | 124 | 25 | 25 |
| 5 | 96 | 18 | 23 |
| 10 | 112 | 16 | 16 |
| 25 | 100 | 17 | 17 |
| 50 | 118 | 12 | 12 |
| **LC-HRMS/MS analysis** | | | |
| 1 | 96 | 16 | 16 |
| 2 | 94 | 11 | 11 |
| 10 | 109 | 9.8 | 14 |
| 100 | 89 | 5.1 | 14 |

**Table S2** Overview of the methods for the determination of hair cortisol in non-domestic animals.

| **Involved animals (number^§^)** | **Investigated variables** | **Hair sampling locations** | **Homogenization** | **Washing** | **Extraction** | **Technique** | **LOD**  **(pg mg^-1^)** | **Detected concentration (min – max or mean – SD – mean + SD)**  **(pg mg^-1^)** | **Reference** |
| --- | --- | --- | --- | --- | --- | --- | --- | --- | --- |
| Rhesus macaques (20) | relocation, washing procedure | posterior vertex region of the neck | grinding | isopropanol  (twice) | MeOH | EIA | 0.9 | 32.1 – 254.3 | Davenport et al. 2006 [20] |
| Grizzly bears (151) | age, body region, capture method, distribution along the hair, hair color, hair type, seasonality, sex, washing procedure | abdomen, neck, rump, shoulder | grinding | MeOH  (five times) | MeOH | EIA | 0.32 | 0.62 – 43.33 | Macbeth et al. 2010 [31] |
| Caribou (12), reindeer (12) | ACTH treatment, body region, sex, subspecies | neck, shoulder, rump | grinding | MeOH (three times) | MeOH | ELISA | 0.32 | NP** | Ashley et al. 2011 [42] |
| Polar bears (17) | age, cross-contamination, sex, washing procedure, year of kill | chest | grinding | isopropanol (twice, three times) | MeOH | EIA | NP | 3.0 – 19.9 | Bechshøft et al. 2011 [53] |
| Dairy cows (83) | body condition score, management, milk yield, seasonality, somatic cell count | forehead | NP | isopropanol (once) | MeOH | RIA | 0.21 | 2.1 - 2.9 | Comin et al. 2011 [19] |
| Dairy cattle (15) | age, hair color, storing condition of sample, treatment (ACTH, saline) | coastal region | cutting (2 mm) | ethanol (once) | MeOH | RIA | NP* | 10.3 – 128.9 | González-de-la-Vara et al. 2011 [64] |
| Polar bears (185) | capture period, family group status, sex | abdomen, neck, rump, shoulder | grinding | MeOH  (three times) | MeOH | EIA | 0.16 | 0.16 – 2.26 | Macbeth et al. 2012 [69] |
| Friesian dairy cows (475) | state of health | forehead | NP | isopropanol (once) | MeOH | RIA | 0.21 | 0.76 - 28.95 | Comin et al. 2013 [70] |
| Asiatic black bears (52) | distribution along the hair, relocation from bile farm, sex, state of health | abdomen | grinding | isopropanol (three times) | Ethanol | EIA | NP | 6.27 – 44.58 | Malcolm et al. 2013 [71] |
| Beef cattle (12) | body region,  collection method | head, hip, neck, shoulder, tail | grinding | isopropanol (twice) | MeOH | EIA | 0.031 | 0.30 – 5.31 | Moya et al. 2013 [72] |
| Dairy cows (290) | breed (purebred/crossbred) | forehead | cutting | isopropanol (once) | MeOH | RIA | NP* | 1.91 – 41.74 | Peric et al. 2013 [21] |
| Lynx (3) | age, body region, sex | foot, hind leg | cutting (5 mm) | MeOH (once) | MeOH | EIA | NP | NP** | Terwissen et al. 2013 [22] |
| Chimpanzees (25) | age, behavior (aggressive – normal), body region, hair color, hair length, institutions, sex | arms, back, sides | grinding | isopropanol (three times) | MeOH | ELISA | NP | NP** | Yamanashi et al. 2013 [23] |
| Sows (30) | delivery, pregnancy, seasonality, weaning time | rump | grinding | water (once), isopropanol (twice) | MeOH | RIA | NP* | 4.66 – 21.05 | Bacci et al. 2014 [24] |
| Lactating dairy cows (18) | body region,  grinding method, hair color | hip, shoulder,  tail switch, top line | grinding | isopropanol (twice) | MeOH | ELISA | 0.03 | 2.7 – 12.2 | Burnett et al. 2014 [25] |
| Brown bears (486) | age, body condition, body mass, capture method, collection method, contour length, dependent offspring, location, sex, time | shoulder | grinding | MeOH (three times) | MeOH | ELISA | 0.32 | 0.94 – 6.58 | Cattet et al. 2014 [26] |
| Rhesus monkeys (152) | age, dominance status, population density, sex | back of the neck | grinding | isopropanol (twice) | MeOH | EIA | NP | 27.95 – 185.30 | Dettmer et al. 2014 [27] |
| Sheeps (9) | heat, water restriction | posterior vertex region of the neck | cutting, grinding | isopropanol (twice), MeOH (twice) | MeOH | ELISA | 0.03 | 0.25 – 2.78 | Ghassemi Nejad et al 2014 [28] |
| Pigs (24) | conventional, transgenic | rump | grinding | water, isopropanol (NP) | MeOH | RIA | 0.21 | 2.14 – 5.96 | Martelli et al. 2014 [29] |
| Chipmunks (12) | age, body condition index, density population, habitat (natural, logged), sex, treatment (ACTH, saline) | hindlimb | cutting (5 mm) | MeOH (once) | 20% Water in MeOH | EIA | 0.0454 | 40.27 - 1050.04 | Mastromonaco et al. 2014 [30] |
| Chimpanzees (48) | body region, distribution along the hair | right and left dorsal forearm, back, right and left shoulder, chest | mincing (3–5 mm) | isopropanol (twice) | MeOH | ELISA | NP | 1.68-25.10 | Carlitz et al. 2015 [32] |
| Lambs (27) | body region, ovine footrot | metatarsus | NP | n-hexane (once) | MeOH | EIA | NP | NP** | Stubsjøen et al 2015 [33] |
| Dairy cows (17) | milking period | forehead, crest | mincing (< 2 mm) | isopropanol (three times) | MeOH | ELISA | 0.32 | 0.67 – 5.34 | Tallo-Parra et al. 2015 [34] |
| Cow (25) | amount, quantitation approach | shoulder | grinding | water (once), acetone (once) | MeOH | LC-MS/MS | 0.5 | NP** | Binz et al. 2016 [35] |
| Baboons (5) | age, body region, life condition (wild-captive), sex | base of the tail, deltoid, thigh | mincing (1-2 mm) | isopropanol (twice) | MeOH | ELISA | 1 | NP** | Fourie et al. 2016 [36] |
| Rabbits (19) | relocation | thigh | cutting | isopropanol (once) | MeOH | RIA | NP* | 1.02 - 3.16 | Peric et al. 2016 [37] |
| Sheep (8) | treatment (extensive brushing, adminstration of hyperemising fluid, dexametasone ) | back | cutting (<5 mm) | n-hexane | MeOH | EIA | NP | NP | Salaberger et al. 2016 [38] |
| Dairy cows (25) | hair growth, illness, pregnancy, seasonality | caudal thorax, cranial thorax, flank, neck, pelvic region, shoulder | cutting (0.5 mm) | water, acetone (once) | MeOH | LC-MS/MS | 0.2/0.1 | 0.07 – 2.50 | Braun et al. 2017 [39] |
| Pigs (49) | body region | craniodorsal, dorsolumbar area | mincing (3 mm) | isopropanol (twice) | MeOH | EIA | 0.17 | 6.40 – 43.88 | Casal et al. 2017 [40] |
| Dairy cows (27) | management | forehead | cutting | isopropanol (once) | MeOH | RIA | NP* | NP** | Peric et al. 2017 [41] |
| Hares (120) | age, breeding technology, sex | sulcus jugularis | grinding | isopropanol (once) | MeOH | ELISA | NP | 6.1 – 27.2 | Esposito et al. 2017 [43] |
| Dairy cows (45) | coat type, hair color, heat | forehead | grinding | isopropanol (twice) | MeOH | EIA | NP | 10.1 – 14.5 | Ghassemi Nejad et al. 2017 [44] |
| Grizzly bears (4),  polar bears (4) | washing procedure, ELISA kit | guard hair | grinding | MeOH, isopropanol  (once-eight times) | MeOH | ELISA | 0.32 | 0.12 – 12.77 | Kroshko et al. 2017 [45] |
| Marmots (8), minks (11) | body region, external arsenic application, seasonality | back, chest, forelimb, hindlimb, rump | cutting (< 5 mm) | MeOH (once) | MeOH | EIA | NP | NP** (marmots)  1.23 – 2.42 (mean values, minks) | Acker et al. 2018 [46] |
| Pigs (65) | coughing, lameness, scouring | rump area | grinding | isopropanol (undefined number of times) | MeOH | EIA | 0.07 | 11.10 – 60.80 | Carrol et al. 2018 [47] |
| Pigs (107) | slaughtering | dorsal area of the neck | grinding | isopropanol (twice) | MeOH | RIA | NP* | 4.45 – 9.06 (mean values) | Bergamin et al. 2019 [48] |
| Veal calves (50) | welfare production labelling | forehead | cutting | water, acetone (once) | MeOH | LC-MS/MS | 0.2/0.1 | 0.69 – 11.55 | Braun et al. 2019 [49] |
| Captive mountain goats (4) | age, hair type (guard, undercoat), reproductive status, sex | rump | cutting (5 mm) | MeOH (once) | 20% Water in MeOH | EIA | NP | NP** | Dulude-de Broin et al. 2019 [50] |
| Sheep (33) | age, body mass, body region, distribution along the shaft, washing procedure | back, shoulder | cutting | MeOH (twice) | MeOH | EIA | NP* | NP** | Fürtbauer et al. 2019 [51] |
| Dairy cows (47), heifers (23) | body region | forehead, withers, rump | cutting | isopropanol (twice) | MeOH | EIA | NP | NP** | Ghassemi Nejad et al. 2019 [52] |
| Sheeps (9), dairy cows (18), heifers (12) | analytical technique, hot and humid environmental conditions | forehead | NP | isopropanol (three times) | MeOH | ELISA RIA | 0.03 (ELISA)  0.03 (RIA) | 0.3 – 9.0 | Ghassemi Nejad et al. 2020 [54] |
| Sheep (46) | pregnancy | shoulder, top knot | cutting (5 mm) | 90% isopropanol in water | 90% ethanol in water | EIA | NP | 0.035 - 5.66 | Sawyer et al. 2019 [55] |
| Cows (540) | dehydration, dung lying in the sheds, injuries on the joints and body, little access to the yards, low body hair loss level, low dry bulb temperature, old age | tail | grinding | water (twice),  isopropanol (once) | MeOH | EIA | NP* | NP | Sharma et al. 2019 [56] |
| Pigs (75)  Cattle (72) | age, age of hair segment, body region, hair color, seasonality, sex | back, neck, shoulder, tail tip | grinding | isopropanol (twice) | MeOH | ELISA | 1.1 | 5.9 – 108.5 | Heimburge et al. 2020 [57] |
| Sows (16) | reproductive cycle | rump | grinding | isopropanol (twice) | MeOH | CLIA (AlphaLISA^®^) | 6.96 (LOQ) | 20.9 – 75.6 | López-Arjona et al. 2020 [58] |
| Dairy cows (21) | alpine grazing | forehead | NP | NP | NP | ELISA | NP | NP** | Ghassemi Nejad et al. 2021 [59] |
| Zoo-housed polar bears (13) | analytical technique, body region, sample homogenization, sampling time, seasonality | front leg (guard hair, undercoat), neck | cutting (≤ 4 mm), mincing | isopropanol (twice) | MeOH | EIA  CLIA  LC-MS/MS | 1.09 (CLIA)  0.072 (EIA)  0.13 (LC-LRMS/MS) | 0.19 – 26.12 | Hein et al. 2021 [60] |
| Sows (251) | reproductive performance, skin lesions | dorso-lumbar | grinding | isopropanol (twice) | MeOH | EIA | NP | NP | Lagoda et al. 2021 [61] |
| Sows (77) and their piglets (997) | farrowing crates (standard, modified), gestation, lactaction, restraint period | rump | grinding | isopropanol (twice) | MeOH | ELISA | NP | 36.3 – 42.7 | Morgan et al. 2021 [62] |
| Pigs (18)  Cattle (18) | artificial light irradiation (black, white color) | back, neck | grinding | isopropanol (twice) | MeOH | ELISA | 0.8 (cattle)  1.1 (pigs) | NP** | Otten et al. 2021 [63] |
| Beef calves (12) | fetal growth | forehead | cutting | isopropanol (twice) | MeOH | RIA | NP* | 2.0 – 31.4 | Probo et al. 2021 [65] |
| Dairy cows (418) | hair color, pregnancy, protein concentration, sampling year, seasonality | rear belly | grinding | isopropanol (twice) | MeOH | RIA | NP | 8.49 – 54.06 | Shi et al. 2021 [66] |
| Sheep (12) | administration of cortisol | NP | grinding | isopropanol (twice) | MeOH | RIA | NP* | 3.1 – 12.9 | Weaver et al. 2021 [67] |
| Sows (31) | body weight loss, farrowing system, number of piglets born alive, number of weaned piglets, parity, skin lesion | neck | NP | isopropanol (twice) | MeOH | CLIA | NP | 0.49 – 8.92 | Wiechers et al. 2021 [68] |

Abbreviations: NP: not provided; EIA: enzyme immunoassay; ELISA: enzyme-linked immunosorbent assay; RIA: radioimmunoassay; CLIA: chemiluminescence immunoassay; AlphaLISA^®^: amplified luminescent proximity homogenous assay; LC-MS/MS: liquid chromatography tandem low resolution mass spectrometry.

^§^Maximum number of animals enrolled in the experiments.

*LOD expressed as sensitivity in solvent.

**Some or all the detected concentrations were only reported in the graphs.
